# Intrahepatic biliary cystadenoma, a challenging diagnosis and management: A case report

**DOI:** 10.1016/j.ijscr.2024.109454

**Published:** 2024-02-28

**Authors:** Yassine Tlili, Zied Hadrich, Montacer Hafsi, Dhouha Bacha, Hafedh Mestiri, Omrani Sahir

**Affiliations:** Department of General Surgery, Mongi Slim University Hospital, Faculty of Medicine of Tunis, Tunisia

**Keywords:** Biliary cystadenoma, Malignant transformation, Recurrence, Radical resection, Ovarian-like stroma

## Abstract

**Introduction and importance:**

Biliary cystadenoma (BC) is a benign hepatic cystic tumor with degenerative potential. Hepatic MRI can help guide the diagnosis. Surgical resection is recommended due to the malignant potential of biliary cystadenomas. Only anatomopathological examination of the surgical specimen can establish the definitive diagnosis of BC. The objective of this case report is to enhance our understanding of this disease and contribute to precise diagnosis for optimal management.

**Case presentation:**

A 55-year-old woman with a history of hypertension and atrial fibrillation presented to the surgery department with paroxysmal right hypochondrial pain. Ultrasonography (US), computed tomography (CT), and magnetic resonance imaging (MRI) revealed a large septated cystic lesion occupying segments II, III, and IV of the liver. The patient underwent left hepatectomy without incident. The postoperative course was marked by a deep collection opposite the sectional area, which was successfully treated with antibiotics and radiological drainage. The pathological diagnosis confirmed BC without signs of malignancy, and no recurrence was detected post-surgery.

**Clinical discussion:**

The rarity of BC, the absence of specific clinical signs and its potential for malignant transformation, underline the need for sophisticated imaging techniques. However, preoperative radiological diagnosis does not exceed 50 %. The operative decision requires a multidisciplinary discussion between radiologists and surgeons. This case highlights the unavailability of radical surgical treatment in cases of strong preoperative suspicion of BC. The cooperation of the pathologist in the histological diagnosis is crucial.

**Conclusion:**

The diagnosis of BC should be considered in cases of multilocular cystic lesions in the liver, particularly in instances of recurrent cysts. Imaging aids in both positive and differential diagnoses. Complete resection is the recommended treatment for any suspected BC.

## Highlights

• Rare benign tumor with malignant potential
• Case presentation and diagnostic techniques
• Multidisciplinary approach and postoperative management
• Importance of imaging in diagnosis and treatment planning

## 1 Introduction

Biliary cystadenoma (BC) is a rare benign cystic tumor of the liver. However, it has a potential of degeneration [1].

Its incidence is estimated at one in 100,000 people [20]. In our practice within the surgical department of Mongi Slim Hospital, one to two cases of biliary cystadenoma are reported per year.

It originates from the bile duct epithelium, and the majority of BCs are intrahepatic [2].

Due to the absence of specific clinical signs, its discovery is most often incidental on radiological examination performed for an unclear abdominal symptomatology, or on pathological examination of resected hepatic cyst [1].

The malignant potential and the recurrence of biliary cystadenomas make complete surgical resection the treatment of choice [3].

The aim of this case report is to deepen our understanding of this disease and contribute to an exact diagnosis in order to optimize its management.

This work was reported in line with the SCARE criteria [19].

## 2 Observation: a case study

A 55-year-old woman, with a history of hypertension and atrial fibrillation under treatment, presented to surgery department suffering from paroxysmal right hypochondrial pain. On examination she had no jaundice neither fever. Abdominal examination was normal. Laboratory tests showed a normal hepatic function.

**Fig. 1:**
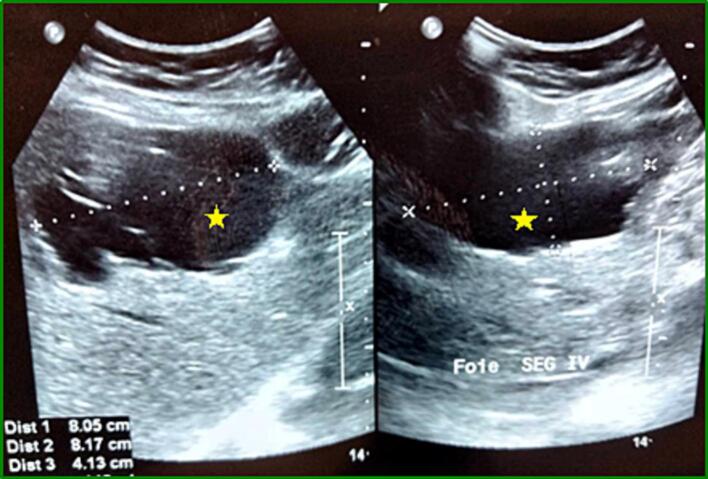
US showing a large anechoic lesion, containing septa and occupying segments II, III and IV of the liver (*).

The trans-abdominal US revealed a large anechoic lesion (8.6 * 6 cm), occupying segments II, III, and IV of the liver, with a thin, regular wall, containing some septa, not vascularized on color Doppler (Fig. 1).

**Fig. 2:**
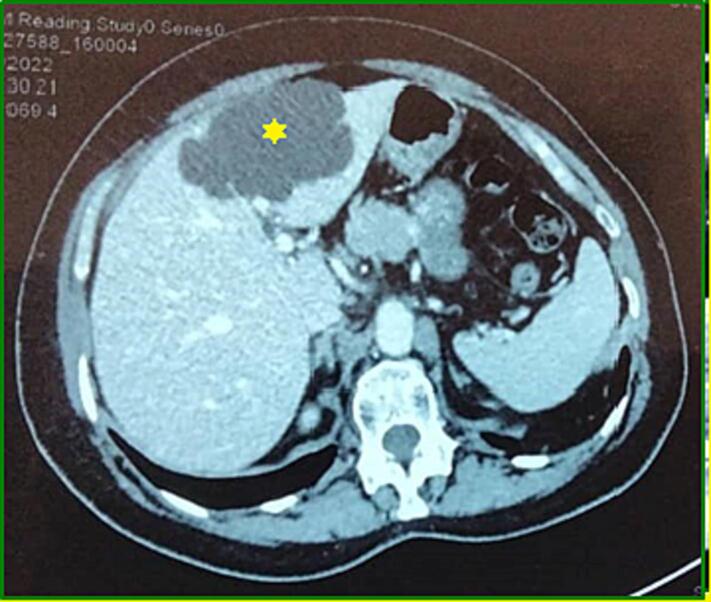
Axial section of an injected CT revealing a fluid-dense hepatic cystic lesion that does not enhance after contrast injection (*).

On abdominal CT, the cyst was partially septated, liquid-dense, with no discernible wall or enhancement after injection of contrast product (Fig. 2).

**Fig. 3:**
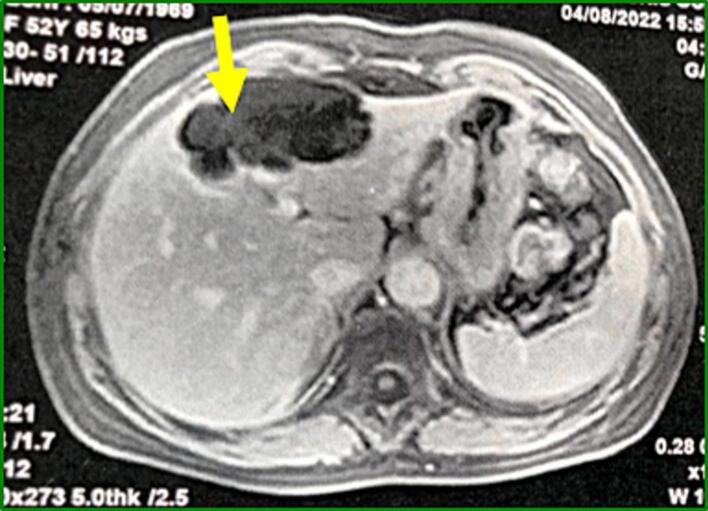
T1-weighted sequences of hepatic MRI revealing a hypointense, fluid-dense mass without clean wall and dotted by fine septa (yellow arrow).

Hepatic MRI was performed to better characterize the lesion. It was a mass with fluid content, T2-hyperintense and T1-hypointense without diffusion restriction, straddling segments II, III and IV. It is the seat of fine septa with no detectable clean wall and no enhancement after gadolinium injection (Fig. 3).

Percutaneous biopsy was not possible due to the risk of rupture.

In light of these results and the risk of malignancy, the operative decision was made. She was approached via Makuuchi incision.

**Fig. 4:**
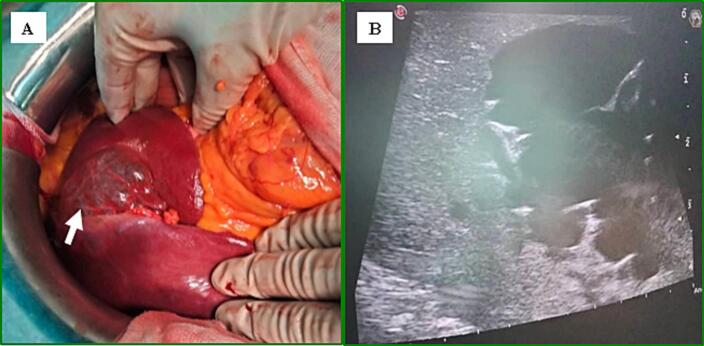
Intraoperative findings: A: Intraoperative view showing the cystic lesion (white arrow); B: multilocular appearance of the lesion on intraoperative US.

Intraoperatively, a thin-walled, cystic lesion of the left liver was discovered (Fig. 4-A). On intraoperative US, the cyst appeared multi-compartmental (Fig. 4-B).

**Fig. 5:**
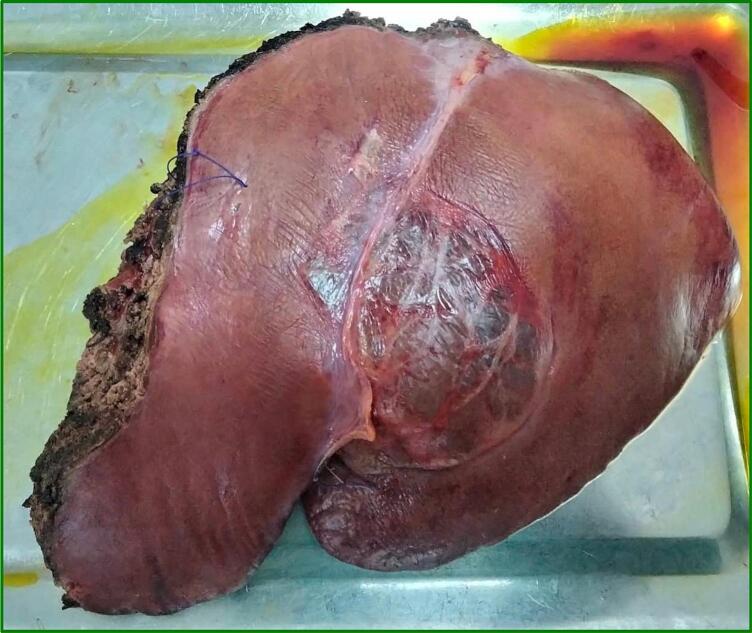
Specimen of left liver resection removing the cystic lesion.

A left hepatectomy was performed without incident (Fig. 5). Drainage was done opposite the hepatic section zone.

Postoperative course was marked by a deep collection opposite the sectional area, which was treated with antibiotics and radiological drainage.

**Fig. 6:**
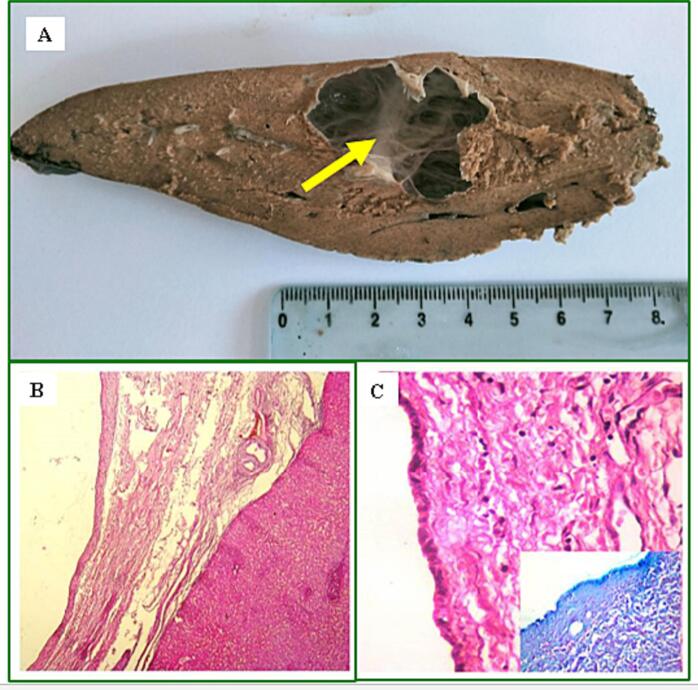
Histopathological findings: A: Multilocular appearance of the cystic mass, without endogenous buds, on cross-section (yellow arrow); B + C: under the microscope, the cyst wall was lined with a single layer of cuboidal/flattened epithelium, with no cyto-nuclear atypia. Ovarian-like stroma was present locally.

The histopathological examination revealed a thin-walled, multilocular cystic formation with clear liquid content upon sectioning (Fig. 6-A). No endocystic buds were observed. The cyst wall is lined by a single layer of epithelium coating, in some areas, an ovarian-like stroma (Fig. 6-B). The cells exhibit cuboidal or flattened morphology, with eosinophilic cytoplasm and no cyto-nuclear atypia (Fig. 6-C). Endocystic papillary projections are absent. The morphological appearance is consistent with a 07 cm long biliary cystadenoma, showing no signs of malignancy.

The patient has no symptoms and no recurrence after three years of follow-up.

## 3 Discussion

Biliary cystadenoma is a very rare, benign cystic tumor of the liver, which accounts for less than 5 % of all hepatic cystic lesions [4].

Despite being classified as benign cystic liver tumors, biliary cystadenomas present a high risk of transformation into biliary cystadenocarcinoma, which can reach 30 % [5].

Historically, the first case was reported by Hueter in 1887, and five years later Keen performed the first resection of a BC [6].

Biliary cystadenomas originate from the biliary epithelium. They are intrahepatic in 85 % of cases, extrahepatic rarely, and their localization in the gallbladder is exceptional [2]. Etiology of cystadenomas remains unclear, but it may be associated with residual foregut or gonadal epithelial tissues in the liver, stemming from abnormal embryonic development [7].

Biliary cystadenomas are more prevalent in women, with a median age of 50. These epidemiological trends are probably attributable to hormonal dependence [2].

BCs are characterized by slow progression and can reach a size of 35 cm [2,8].

Most patients are asymptomatic. Nonspecific symptoms may be present, such as abdominal pain, abdominal distension, abdominal mass or vomiting. Jaundice may be observed in cases of large biliary cystadenomas exerting a mass effect on the main bile duct [9]. Unusual complications of biliary cystadenomas were described in the literature, such as intracystic hemorrhage, infection and rupture [10].

BCs are often multilocular. Unilocular forms are exceptional and may be confused with simple biliary cysts [2]. The majority of BCs have no communication with the bile ducts, although rare cases may involve biliary communication [11].

Biologically, liver function tests are often normal, and there are no specific tumor biomarkers. However, elevated CA19–9 levels have been observed in 60 % of patients with BC and biliary cystadenocarcinoma, and these levels return to normal after complete cyst resection. CA19–9 levels are thus considered a diagnostic and prognostic element [12]. Detection of intracystic CA19–9 via cyst puncture is controversial and not standard practice. Indeed, cyst puncture is associated with a risk of bleeding and tumor dissemination [13].

The radiological diagnosis of biliary cystadenomas is challenging. Several cystic pathologies of the liver may be confused with BC, including simple liver cyst, hydatid cyst, polycystic liver disease, cystadenocarcinoma… [2].

Typically, BC presents as a multiloculated cyst, dotted with septa and occasionally containing non-contrast-enhancing mural nodules [2]. Color Doppler US is more sensitive in detecting septa within cystic lesions, while CT reveals the size and the anatomic relation to vasculo-biliary structures [14]. MRI offers better diagnostic value, and the classic biliary cystadenoma presents as a multilocular cyst with an irregular wall and heterogeneous signal. It is hypointense on T1-weighted sequences and hyperintense on T2-weighted sequences; however, signal intensity may be influenced by the properties of the cystic fluid [15]. Contrast-enhanced US is a useful radiological examination for the diagnosis of hepatic cystic lesions, in particularly biliary cystadenoma [16].

Significant thickening of the septa, contrast-enhancing papillary protrusions or wall nodules, macroscopic calcification or intracystic hemorrhage indicate cystadenocarcinoma [14].

Despite the use of sophisticated techniques, the preoperative radiological diagnosis of biliary cystadenomas is erroneous in over 50 % of cases, underlining the need for a high index of suspicion [2]. To minimize diagnostic errors, liver cysts recurring after fenestration should be regarded as biliary cystadenomas, and a systematic intraoperative frozen section examination should be conducted for all operated simple liver cysts [14].

A preoperative biopsy or fine-needle aspiration cytology to detect malignancy is not recommended, particularly for operable lesions, due to the lack of precision and the risk of tumor spread [13].

The definitive diagnostic method is the anatomopathological examination of resected cysts. Histologically, BC exhibit a wall lined by a simple epithelium overlying a stroma that may resemble ovarian tissue. BC lacking ovarian-like stroma carry an increased risk of malignant transformation and have a less favorable prognosis [17].

Complete surgical resection of BCs is the treatment of choice, ensuring a favorable long-term prognosis (less than 10 % of recurrence). Incomplete resection is associated with a high risk of recurrence (exceeding 80 %) and malignant transformation [18,20]. Enucleation of BCs is a valid alternative when resection is difficult or is likely to be associated with high morbidity [2]. Surgical exeresis of centrohepatic cystadenomas requires discussion between radiologists, surgeons and anaesthetists, in order to interpret the therapeutic possibilities in terms of vascular-biliary connections and the patient's general condition.

Post-operative follow-up is recommended to detect short-term (deep collection, biliary fistula) and long-term (recurrence, malignant transformation) post-operative complications. The procedure adopted in our department following hepatectomy for BC is as follows:

- Clinical check-up at 15 days post-operatively
- Clinical check-up combined with liver function tests at 1 month.
- Clinical check-up, liver function tests and abdominal CT scan at 6 months.
- Clinical check-up at 1 and 2 years post-operatively
- Abdominal CT scan at 3 years post operatively.

This frequency of surveillance is subject to adaptation according to warning signs and complications.

## 4 Conclusion

This article highlights a case of biliary cystadenoma, the diagnosis of which was suspected by a range of radiological examinations.

The diagnosis of BC should be considered in cases of multilocular cystic lesions in the liver, particularly recurrent cysts. Imaging facilitates positive and differential diagnosis.

The recommended treatment for any suspected BC is complete resection, as it is extremely difficult to distinguish it from cystadenocarcinoma preoperatively.

The study of our observation underlines two rules in the management of cystic lesions of the liver: any suspicious hepatic lesion must be completely resected, and confirmation of the diagnosis is always histological.

## Consent for publication

Written informed consent was obtained from the patient for publication of this case report and any accompanying images. A copy of the written consent is available for review by the Editor-in-Chief of this journal.

## Ethical approval

Not applicable. Our institution requires no ethical approval for case reports.

## Funding

Not applicable.

## Guarantor

The corresponding author is the guarantor of submission.

## Research registration number

1. Name of the registry: none
2. Unique identifying number or registration ID: none
3. Hyperlink to your specific registration (must be publicly accessible and will be checked): –.

## CRediT authorship contribution statement

Yassine Tlili: conception and design, acquisition of data drafting the article, revising it critically for important intellectual content final approval of the version to be published
Zied Hadrich: conception and design, acquisition of data drafting the article, revising it critically for important intellectual content final approval of the version to be published
Montacer Hafsi: conception and design, acquisition of data drafting the article, revising it critically for important intellectual content final approval of the version to be published
Dhouha Bacha: final approval of the version to be published
Hafedh Mestiri: final approval of the version to be published
Sahir Omrani: final approval of the version to be published.

## Declaration of competing interest

All authors declare that they have no conflicts of interest.

## Data Availability

Not applicable.

## References

[1] Xu R.M., Li X.R., Liu L.H., Zheng W.Q., Zhou H., Wang X.C. (2020). Intrahepatic biliary cystadenoma: a case report. World J. Clin. Cases.

[2] Sharma D., Gondu G.R., Thamma V.M., Gunturi S.R.V., Kishore K.N., Reddy J.M. (2020). Recurrent giant intrahepatic biliary cystadenoma: case report and review of literature. Indian J Med Paediatr Oncol..

[3] Hernandez Bartolome M.A., Fuerte Ruiz S., Manzanedo Romero I., Ramos Lojo B., Rodriguez Prieto I., Gimenez Alvira L. (2009). Biliary cystadenoma. World J. Gastroenterol..

[4] Emre A., Serin K.R., Ozden I., Tekant Y., Bilge O., Alper A. (2011). Intrahepatic biliary cystic neoplasms: surgical results of 9 patients and literature review. World J. Gastroenterol..

[5] Delis S.G., Touloumis Z., Bakoyiannis A., Tassopoulos N., Paraskeva K., Athanassiou K., Safioleas M., Dervenis C. (2008). Intrahepatic biliary cystadenoma: a need for radical resection. Eur. J. Gastroenterol. Hepatol..

[6] Short W.F., Nedwich A., Levy H.A., Howard J.M. (1971). Biliary cystadenoma. Report of a case and review of the literature. Arch. Surg..

[7] Treska V., Ferda J., Daum O., Liska V., Skalicky T., Bruha J. (2016). Intrahepatic biliary cystadenoma - diagnosis and treatment options. Turk J Gastroenterol.

[8] Soares K.C., Arnaoutakis D.J., Kamel I., Anders R., Adams R.B., Bauer T.W., Pawlik T.M. (2014). Cystic neoplasms of the liver: biliary cystadenoma and cystadenocarcinoma. J. Am. Coll. Surg..

[9] Williamson J.M., Rees J.R., Pope I., Strickland A. (2013). Hepatobiliary cystadenomas. Ann. R. Coll. Surg. Engl..

[10] Barabino M., Leone S., Dapri G., Marsetti M., Ghislandi R., Opocher E. (2004). Hepatobiliary cystadenoma: diagnostic uncertainty. HPB.

[11] Del Poggio P., Buonocore M. (2008). Cystic tumors of the liver: a practical approach. World J. Gastroenterol..

[12] Xu M.Y., Shi X.J., Wan T., Liang Y.R., Wang H.G., Zhang W.Z. (2015). Clinicopathological characteristics and prognostic factors of intrahepatic biliary cystadenocarcinoma. Chin. Med. J..

[13] Liang X., Zheng J., Gao J. (2018). Advances in diagnosis and treatment of intrahepatic biliary cystadenoma. Chin J Dig Surg..

[14] Yang Y., Chen W., Cen H. (2022). Intrahepatic biliary cystadenoma: confusion, experience, and lessons learned from our center. Front. Oncol..

[15] Lewin M., Mourra N., Honigman I., Fléjou J.F., Parc R., Arrivé L. (2006). Assessment of MRI and MRCP in diagnosis of biliary cystadenoma and cystadenocarcinoma. Eur. Radiol..

[16] Che C.H., Zhao Z.H., Song H.M., Zheng Y.Y. (2021). Rare monolocular intrahepatic biliary cystadenoma: a case report. World J. Clin. Cases.

[17] Manouras A., Markogiannakis H., Lagoudianakis E., Katergiannakis V. (2006). Biliary cystadenoma with mesenchymal stroma: report of a case and review of the literature. World J. Gastroenterol..

[18] Thomas K.T., Welch D., Trueblood A., Sulur P., Wise P., Gorden D.L. (2005). Effective treatment of biliary cystadenoma. Ann. Surg..

[19] Sohrabi C., Mathew G., Maria N., Kerwan A., Franchi T., Agha R.A. (2023). The SCARE 2023 guideline: updating consensus Surgical CAse REport (SCARE) guidelines. Int J Surg Lond Engl..

[20] Pitchaimuthu M., Aidoo-Micah G., Coldham C., Sutcliffe R. (2015). Outcome following resection of biliary cystadenoma: a single centre experience and literature review. Int J Hepatol..

